# Does getting a dog increase recreational walking?

**DOI:** 10.1186/1479-5868-5-17

**Published:** 2008-03-27

**Authors:** Hayley E Cutt, Matthew W Knuiman, Billie Giles-Corti

**Affiliations:** 1 School of Population Health, The University of Western Australia, Crawley, Western Australia

## Abstract

**Background:**

This study examines changes in socio-demographic, environmental and intrapersonal factors associated with dog acquisition in non-dog owners at baseline to 12-months follow-up and the effect of dog acquisition on minutes per week of recreational walking.

**Methods:**

RESIDE study participants completed self-administered questionnaires (baseline and 12-months follow-up) measuring physical activity, dog ownership, dog walking behavior as well as environmental, intrapersonal and socio-demographic factors. Analysis was restricted to 'Continuing non-owners' (i.e., non-owners at both baseline and follow-up; n = 681) and 'New dog owners' (i.e., non-owners who acquired a dog by follow-up; n = 92).

**Results:**

Overall, 12% of baseline non-owners had acquired a dog at follow-up. Dog acquisition was associated with working and having children at home. Those who changed from single to couple marital status were also more likely to acquire a dog. The increase in minutes of walking for recreation within the neighborhood from baseline to follow-up was 48 minutes/week for new dog owners compared with 12 minutes/week for continuing non-owners (*p *< 0.05). After adjusting for baseline variables the effect of dog acquisition on the increase in minutes of recreational walking within the neighborhood was 31 minutes (95% CI: 7.39, 54.22; *p *< 0.01). However, this reduced to 22 minutes (95% CI: -1.53, 45.42; *p *> 0.05) after further adjustment for change in baseline to follow-up variables. Increase in intention to walk was the main factor contributing to attenuation of the effect of dog acquisition on recreational walking.

**Conclusion:**

This study used a large representative sample of non-owners to examine the relationship between dog acquisition and recreational walking and provides evidence to suggest that dog acquisition *leads *to an increase in walking. The most likely mechanism through which dog acquisition facilitates increased physical activity is through behavioral intention via the dog's positive effect on owner's cognitive beliefs about walking, and through the provision of motivation and social support for walking. The results suggest that behavioral intention mediates the relationship between dog acquisition and walking and that dogs may have a significant role in the maintenance of owner walking behavior.

## Background

Over half of all adults in the United States and Australia do not meet the recommended level of physical activity necessary for health benefit [1,2]. Growing concerns about the level of inactivity has resulted in a recent focus on the effect of the built environment on health [3,4]. However, another new area of physical activity research beginning to leave its paw mark is the association between dog ownership and physical activity. Given the sub-optimal participation in physical activity by most adults, and the high level of dog ownership in the community [5] exploring whether responsible dog ownership could be used as a means to promote more walking is worthy of further investigation.

Cross-sectional studies suggest that dog owners are more physically active than non-owners [6,7] and are more likely to achieve the recommended level of activity [8-11], even after adjustment for socio-demographic, intrapersonal, social environmental and physical environmental confounders [12]. However, some studies have emphasized that dog ownership does not necessarily equate to dog walking as up to 60% of dog owners do not walk with their dog [8]. We recently showed that owners were less likely to walk with their dog if they did not perceive that their dog provided social support or motivation to walk more [13]. Considering almost 40% of households own a dog, there would be a significant impact on community physical activity levels if all dog owners were physically active.

Evidence of the potential for dog ownership to facilitate higher levels of physical activity has been limited to date because it is mainly cross-sectional. One small study of adults who acquired a pet (dog or cat) from an animal shelter (n = 71) examined whether pet acquisition changed owner's health status, including their physical activity. Compared with cat owners and the control group, dog owners increased and maintained their walking from one hour/week at baseline to five hours/week at 10 months follow-up [14,15]. While these findings are promising, the study had limitations. First, people who acquire a dog from an animal shelter may not be representative of other dog owners and dogs acquired from animal shelters may not be representative of all dogs. Second, this study had a small sample size and a relatively short follow-up period. Finally, the study did not control for other factors associated with acquiring a dog.

Only two prospective studies have examined the association between dog ownership and physical activity over time and both of these studies were conducted in an elderly population [11,16]. Thorpe and colleagues reported that at three years follow up, dog walkers maintained their initial mobility advantage (i.e. higher walking speed) over other dog ownership and walking-status groups [11]. These results support the notion that dog walkers maintain their health advantage over time however causality cannot be implied because change in dog ownership status was not examined. Importantly, does dog ownership lead to an increase in physical activity or are active individuals more likely to own a dog? Moreover, what factors associated with dog acquisition need to be adjusted for when examining the temporal relationship between dog acquisition and physical activity? Thus, the first aim of this study was to identify baseline socio-demographic, intrapersonal and physical and social environmental factors associated with dog acquisition. The second aim was to examine longitudinal (baseline to follow-up) changes in these factors and their association with dog acquisition. The third aim was to examine the effect of dog acquisition on recreational walking and identify confounders and mediators of the effect of dog acquisition on changes in recreational walking.

## Methods

### Sample and procedure

The sample included all baseline non-dog owners taking part in the RESIDential Environments (RESIDE) project, a 5-year longitudinal study evaluating the impact of a state-government sub-division code in Perth, Western Australia [17]. Described fully elsewhere [18], RESIDE involves new home owners self-completing a questionnaire before they move into their new home (n = 1813), then 12 (n = 1379) and 36 months later, after moving into their new home. All people building new homes in the study area were invited to participate and those agreeing to take part provided written informed consent (response rate 33.4%). RESIDE participants who completed a baseline questionnaire between September 2003-March 2005 completed a second questionnaire approximately 12 months later at first follow-up (October 2005-December 2006). This study was approved by The University of Western Australia's Human Research Ethics Committee.

### Dog acquisition

At both time points participants were asked about the type of pet(s) owned. The responses included dog, cat, bird and other pet. Only baseline non-dog owners who at 12 months follow-up either remained non-owners or had acquired a dog were included in the study (n = 773). Participants' who did not own a dog at baseline but who had acquired a dog at follow-up were classified as 'New dog owners' while participants who were non-dog owners at baseline and follow-up were classified as 'Continuing non-owners'.

### Self-reported physical activity, walking and dog walking

Self-reported physical activity over a usual week was collected using the Neighborhood Physical Activity Questionnaire (NPAQ), which differentiates between walking within and outside of the neighborhood and has acceptable reliability [17]. NPAQ measures of physical activity included total minutes per usual week of: 1) physical activity; 2) walking; and, 3) walking for recreation in the neighborhood. Minutes of dog walking/usual week were collected using the Dogs and Physical Activity (DAPA) tool [19]. The DAPA tool is reliable and has face and construct validity [19].

### Covariates

Baseline socio-demographic variables included: gender, age, country of origin, marital status, presence of children <18 years at home, mean age of children <18 years at home, education level attained, work status, number of hours worked/week, occupational status, household income and type of residence. New categorical socio-demographic variables were created to reflect changes in these variables between baseline and follow-up.

A modified version of the Neighborhood Environment Walkability Scale (NEWS) [20] was used to measure perceptions of the physical environment. Perceptions of social support from family and friends for walking and other physical activity were measured using modified items of the social support for exercise questions developed by Sallis and others [21]. The Neighborhood Cohesion Scale [22] was used to measure community cohesion. Measures of intention, attitude toward the process of trying, perceived behavioral control, self-efficacy and behavioral skills were assessed using standard items reported previously [23,24] and the enjoyment of walking variable was adapted from the Physical Activity Enjoyment Scale [25]. Further detail of these scales have been described elsewhere [12,18]. Changes in physical environmental (land use mix, aesthetics, walking facilities, street connectivity and safety), social environmental (social support and neighborhood social cohesion) and intrapersonal (intention, enjoyment, attitude, self-efficacy, perceived behavioral control and behavioral skills) scales between baseline and follow-up were calculated.

Most change variables were coded as no change, increase or decrease from baseline to follow-up. Four socio-demographic change variables were coded differently: marital status (no change, couple to single, single to couple); work status (no change, now in workforce, no longer in workforce); children under 18 years at home (no change, children now living at home, children no longer living at home); and type of residence (no change, moved to a separate house). For example, no change in marital status included participants who were the same marital status [either single (separated/divorced, widowed or single/never married) or couple (married or defacto)] at both time points.

### Statistical analysis

Chi square and independent sample t-tests were used to examine the association between dog acquisition and, baseline socio-demographic and change from baseline to follow-up variables. Those variables found to be significant at *p *≤ 0.05 were entered (forced entry) into a linear regression model to investigate the association between dog acquisition and change in minutes of recreational walking in the neighborhood. Three models were constructed. The first model was unadjusted; the second model adjusted for baseline recreational walking in the neighborhood and significant baseline socio-demographic variables from Table 1 and the third model further adjusted for significant change from baseline to follow-up variables from Tables 2 and 3. Mediation analysis was undertaken on the final model by removing significant variables and observing the change in the effect of dog acquisition on increase in minutes of recreational walking. All models were a complete case analysis (n = 695) with a significance level of *p *≤ 0.05.

**Table 1:** Baseline socio-demographic characteristics of non-owners at baseline by dog ownership status at one year follow-up

**Characteristic**	**% New dog owners Mean (SD) (n = 92)**	**% Continuing non-owners Mean (SD) (n = 681)**	***p *value**
Gender (female)	62.0	57.3	0.395
Mean age (years)	39.1 (SD 9.59)	41.68 (SD 12.48)	**0.021**
Born in Australia	59.3	54.1	0.342
Marital status			
*Married/defacto*	75.0	84.4	**0.037**
*Separated/divorced/widowed*	15.2	7.7	
*Single*	9.8	8.0	
Education			
*Secondary or less*	38.5	38.5	0.073
*Trade/apprentice/certificate*	45.1	35.2	
*Bachelor or higher*	16.5	26.3	
Work status			
*Work*	87.0	77.2	**0.036**
*No work*	12.0	15.2	
*Retired*	1.1	7.7	
Number of hours worked^1^			
≤ *Half time*	15.3 (16.5)	10.2 (13.0)	**0.005**
*>Half time ≤ 38 hrs/week*	23.5 (25.3)	22.8 (29.2)	(0.260)
*>38 hrs/week ≤ 60 hrs/week*	52.9 (57.0)	40.6 (51.9)	
*>60 hrs/week*	1.2 (1.3)	4.6 (5.8)	
*Not in workforce*	7.1	21.8	
Occupation^1^			
*Management/administration*	20.7 (22.2)	12.8 (16.3)	**0.002**
*Professional*	26.4 (28.4)	30.6 (38.8)	(0.111)
*Blue collar*	23.0 (24.7)	13.4 (17.0)	
*Clerical/sales/service/other*	23.0 (24.7)	21.9 (27.8)	
*Not in workforce*	6.9	21.3	
Household income			
*<$49, 999*	23.8	28.1	0.859
*$50–69,999*	27.4	25.3	
*$70–89,999*	22.6	22.4	
*$90,000+*	26.2	24.1	
Children living at home <18 years	61.1	48.4	**0.024**
Mean age of children living at home	7.24 (SD 4.20)	6.43 (SD 4.54)	0.219
<18 years			
Type of residence			
*Separate house*	81.5	80.2	0.652
*Semi-attached*	8.7	9.6	
*Flat/unit*	7.6	9.3	
*Other*	2.2	0.9	

^1 ^Results presented within brackets for number of hours worked and occupation excludes those not in the workforce from the sample

**Table 2:** Change in socio-demographic characteristics for non-owners at baseline by dog ownership status at one year follow-up

**Characteristic**	**% New dog owners % (n = 92)**	**Continuing non-owners (n = 681)**	***p *value**
Marital status			
*No change*	91.2	96.0	**0.028**
*Couple to single*	1.1	1.5	
*Single to couple*	7.7	2.5	
Education status			
*No change*	93.2	93.3	0.960
*Increase*	6.8	6.7	
Work status			
*No change*	92.3	93.0	0.730
*Now in workforce*	3.3	4.0	
*No longer in workforce*	4.4	3.0	
Number of hours worked			
*No change*	63.9	69.0	0.398
*Increase*	13.3	14.1	
*Decrease*	22.9	16.9	
Time to travel work			
*No change*	53.9	64.5	0.148
*Increase*	27.0	20.1	
*Decrease*	19.1	15.4	
Occupation status			
*No change*	72.4	76.6	0.328
*Increase*	11.5	12.7	
*Decrease*	16.1	10.7	
Household income			
*No change*	56.8	62.6	0.227
*Increase*	34.6	25.8	
*Decrease*	8.6	11.6	
Children living at home <18 years			
*No change*	94.2	93.1	0.644
*Children now living at home*	2.3	4.2	
*Children no longer living at home*	3.5	2.7	
Type of residence			
*No change*	81.5	80.2	0.771
*Moved to separate house*	18.5	19.8	

**Table 3:** Change in perceived neighborhood characteristics, perceived social support provided in past month, neighborhood cohesion score and intrapersonal factors for non-owners at baseline by dog ownership status at one year follow-up

**Physical environmental sub-scales:**	**% New dog owners (n = 92)**	**% Continuing non-owners (n = 681)**	***p *value**
Land use mix-access			
*No change*	45.7	48.4	0.596
*Increase*	14.1	10.6	
*Decrease*	40.2	40.9	
Aesthetics			
*No change*	53.8	48.3	0.423
*Increase*	25.3	32.0	
*Decrease*	20.9	19.7	
Walking facilities			
*No change*	57.6	54.7	0.810
*Increase*	22.8	25.9	
*Decrease*	19.6	19.4	
Park or nature reserve that's easily accessible			
*No change*	58.7	53.0	0.582
*Increase*	28.3	31.8	
*Decrease*	13.0	15.2	
Street connectivity			
*No change*	56.5	61.9	0.164
*Increase*	22.8	24.8	
*Decrease*	20.7	13.3	
Pedestrian/traffic safety			
*No change*	53.8	47.7	0.200
*Increase*	24.2	33.5	
*Decrease*	22.0	18.8	
Crime safety			
*No change*	50.0	48.7	0.801
*Increase*	40.2	43.1	
*Decrease*	9.8	8.1	
Design of new neighborhood			
*Conventional*	53.3	51.0	0.913
*Hybrid*	18.5	19.0	
*Liveable*	28.3	30.0	

**Social environmental sub-scales:**			

Social support from family for walking			
*No change*	33.3	37.6	0.244
*Increase*	44.0	34.8	
*Decrease*	22.6	27.6	
Social support from family for walking			
*No change*	57.1	8.8	0.288
*Increase*	17.9	22.7	
*Decrease*	25.0	18.5	
Social support from friends for other physical activity			
*No change*	36.6	41.3	0.684
*Increase*	35.4	33.8	
*Decrease*	28.0	24.8	
Social support from friends for other physical activity			
*No change*	51.2	56.4	0.398
*Increase*	22.0	23.3	
*Decrease*	26.8	20.4	
Neighborhood social cohesion			
*No change*	38.5	47.1	0.154
*Increase*	48.4	44.6	
*Decrease*	13.2	8.3	

**Intrapersonal items and sub-scales:**			

Intention to walk for total 30 mins on ≥ 5 days/week			
*No change*	19.6	31.3	**0.001**
*Increase*	58.7	37.7	
*Decrease*	21.7	31.0	
Intention to do vigorous leisure time physical activity			
for total three 20 min sessions/week			
*No change*	28.3	32.6	0.378
*Increase*	30.4	33.4	
*Decrease*	41.3	34.0	
Intention to do other moderate leisure time physical			
activity for total 30 mins on ≥ 5 days/week			
*No change*	20.7	28.5	0.178
*Increase*	37.0	37.5	
*Decrease*	42.4	34.0	
Enjoyment of walking in neighborhood			
*No change*	60.4	57.6	0.860
*Increase*	16.5	18.5	
*Decrease*	23.1	23.9	
Attitude toward process of trying to walk on most days			
*No change*	40.2	44.6	0.699
*Increase*	39.1	35.2	
*Decrease*	20.7	20.2	
Self-efficacy			
*No change*	40.7	50.2	**0.016**
*Increase*	34.1	20.7	
*Decrease*	25.3	29.0	
Perceived behavioral control			
*No change*	28.3	36.1	0.089
*Increase*	47.8	36.1	
*Decrease*	23.9	27.8	
Behavioral skills			
*No change*	51.1	46.4	**0.049**
*Increase*	30.4	23.3	
*Decrease*	18.5	30.3	

## Results

### Characteristics of people who acquire a dog

Twelve percent (n = 92) of baseline non-dog owners (n = 773) acquired a dog by follow-up. A number of baseline socio-demographic characteristics were significantly associated with dog acquisition (Table 1). At baseline, significantly more non-owners who had acquired a dog by follow-up were separated, divorced or widowed, had children <18 years living at home, were slightly younger and participated in the workforce than continuing non-owners (*p *< 0.05). No significant differences between new dog owners and continuing non-owners baseline physical or social environments were observed (results not shown).

Changes in marital status (Table 2), intention to walk, self-efficacy and behavioral skills (Table 3) significantly differed for new dog owners and continuing non-owners at follow-up. More new dog owners than continuing non-owners moved from a single to couple relationship between baseline and follow-up (*p *< 0.05). Furthermore, more new dog owners than continuing non-owners reported an increase in their intention, self-efficacy and use of behavioral skills for walking in the next month (*p *< 0.05). There were no other significant changes in physical and social environments or individual factors by dog acquisition at follow-up.

### Physical activity behavior of new dog owners and continuing non-owners at baseline and first follow-up

At baseline new dog owners did significantly less average weekly minutes of overall walking than continuing non-owners (89.6 vs. 117.8; *p *< 0.05) (Table 4). Mean weekly minutes of total physical activity and recreational walking in the neighborhood differed for new dog owners and continuing non-owners at baseline but was not statistically significant.

**Table 4:** Baseline mean minutes of physical activity and change in mean minutes of physical activity for non-owners at baseline by dog ownership status at one year follow-up

	**New dog owners: Mean minutes (SEM)**	**Continuing non-owners: Mean minutes (SEM)**	***p *value**
Baseline physical activity	278.37 (35.25)	251.37 (11.94)	0.447
Baseline walking	89.56 (12.16)	117.83 (5.95)	**0.039**
Baseline walking for recreation in neighborhood	44.87 (8.01)	58.16 (3.72)	0.216
Change in physical activity	32.44 (28.04)	2.75 (11.03)	0.357
Change in walking	38.00 (15.30)	-4.75 (6.35)	**0.021**
Change in walking for recreation in neighborhood	48.00 (10.17)	12.13 (4.63)	**0.007**
Walking with dog in neighborhood	130.35 (13.71)	-	-

At follow-up, new dog owners walked with their dog in their neighborhood an average of 130 minutes/week (Table 4). Increase in minutes of walking for recreation within the neighborhood between baseline and follow up totaled 48 minutes/week for new dog owners and 12 minutes/week for continuing non-owners (*p *< 0.05). Moreover, new dog owners increased their total walking by 38 minutes/week between baseline and follow-up. In the same period, continuing non-owners decreased their walking by 5 minutes/week (*p *< 0.05). While new dog owners reported an increase in their overall physical activity of 32 minutes/week, this was not significantly different to that of continuing non-owners (3 minutes/week).

### Does dog acquisition increase recreational walking?

Linear regression models were used to examine the unadjusted and adjusted effects of dog acquisition on change in minutes of recreational walking within the neighborhood. In the unadjusted model (model 1), the increase in minutes/week of recreational walking within the neighborhood was 35.9 minutes (95% CI: 9.65, 62.09; *p *< 0.01) greater in those who acquired a dog compared with continuing non-owners. After adjusting for baseline walking for recreation within the neighborhood and significant baseline socio-demographic variables (model 2), the effect of dog acquisition on the increase in minutes of recreational walking within the neighborhood attenuated to 30.8 minutes but remained statistically significant (95% CI: 7.39, 54.22; *p *< 0.01). Baseline walking for recreation in the neighborhood accounted for almost all of the attenuation. Nevertheless, when the model was further adjusted for significant change in baseline to follow-up variables (model 3), the effect of dog acquisition on the increase in minutes of recreational walking within the neighborhood was reduced to 21.9 minutes and was no longer statistically significant (95% CI: -1.53, 45.42; *p *> 0.05). Further modeling revealed that when the variable for change in intention to walk was dropped from model 3, the effect of dog acquisition on the increase in the minutes of recreational walking within the neighborhood increased to 27.3 minutes and was once again statistically significant (95% CI: 3.71, 50.82; *p *< 0.05) thus indicating that adjustment for change in intention to walk was the principal reason for the reduced effect of dog acquisition. The other three change variables (marital status, self-efficacy and use of behavioral skills for walking in the next month) collectively were responsible for the remaining difference of 3.5 minutes.

## Discussion

The results of this study suggest that dog acquisition leads to a significant increase in recreational walking and the mechanism through which dog ownership increases recreational walking is behavioral intention. Non-owners, who acquired a dog by follow-up, increased their recreational walking by 31 minutes/week more than continuing non-owners even after adjustment for baseline factors associated with dog acquisition. This value is considerably less than the 240 minutes/week increase in walking reported by Serpell [14,15] however, Serpell's study did not control for potential confounders and involved a smaller non-representative sample who were followed for a shorter period of time. In contrast, the current study used a large representative sample of non-owners to examine if dog acquisition leads to an increase in recreational walking and controlled for baseline characteristics.

After adjusting for factors associated with dog acquisition (both baseline and change in baseline to follow-up variables), the increase in minutes of recreational walking reduced from 36 to 22 minutes/week and was no longer statistically significant. Thus, increase in intention to walk was associated with both dog acquisition and increased recreational walking and explains a large part of the effect of dog acquisition on increased recreational walking. While it appears that change in intention to walk is a significant mediator of this relationship, the temporal order of dog acquisition and increased intention to walk is unconfirmed. For instance, does interest, capacity or intention to walk in the next month increase and as a result a dog is acquired to assist in shifting intention to action, or is a dog acquired and through a sense of obligation to care for the dog, intention to walk increases [11]?

Acquiring a dog is coupled with a responsibility to care for the health and well-being of that dog [26]. Basic care for a dog includes the provision of food, water, shelter and exercise [27]. It is likely that through a person's sense of responsibility to care for their dog, cognitive beliefs about providing a safe and healthy environment for a dog may positively influence an individual's intention to walk. Dog owners may feel they have a sense of responsibility to exercise their dog and this increases their own intention to walk. A Canadian study reported that obligation or responsibility to walk a dog mediated the relationship between dog ownership and physical activity and explained 1% of the variance in intention to walk [6]. Moreover, it is also possible that people's intention to walk in the next month increases and they acquire a dog to help turn their intentions into action. However, it seems unlikely that people would acquire a dog purely for the sake of motivating them to exercise [28-31] especially considering that in most instances (85%) the main function of a pet dog is companionship [29,32,33]. Furthermore, even if an individual has an intention to walk and acquires a dog to help realize those intentions, sense of responsibility to care for the well-being of their dog is likely to increase recreational walking more than if an individual has an intention to walk but does not acquire a dog.

In this study, the increase in overall walking was greater than the increase in recreational walking in the neighborhood (43 vs. 36 minutes). Although the increase in minutes of recreational walking associated with dog acquisition represented a significant increase, walking for recreation makes up only one component of all walking. Thus, it is possible that new dog owners also increased their time spent walking for recreation outside their neighborhood (for example, walking at a dog beach) as well as transport-related walking (for example, walking to local shop with dog). Moreover, at baseline new dog owners did significantly less weekly minutes of overall walking than continuing non-owners. This suggests that new dog owners had greater potential than continuing non-owners to increase their weekly minutes of overall walking and may in part explain why the increase in minutes of overall walking was more than the increase in minutes of recreational walking in the neighborhood.

Furthermore, the increase in minutes of recreational walking and overall walking associated with dog acquisition was not reflected in the increase in total physical activity (36 vs. 30 and 43 vs. 30 minutes respectively), suggesting that recreational walking was substituted in place of other types of physical activity. It could be that confounding factors influence the relationship between dog acquisition and change in minutes of total physical activity as was shown for recreational walking. However, it is more likely that new dog owners increased their minutes of recreational walking at the expense of other types of physical activity. In this study, baseline levels of total physical activity were well above the recommended level of 150 minutes/week and there was no significant difference in baseline minutes of total physical activity for new dog owners and continuing non-owners. These results suggest that there was little potential for minutes of total physical activity to increase post dog acquisition and it is likely that new dog owners substituted other types of physical activity for more dog walking.

The Marchetti principle [34] applied in the transport literature suggests that people have an average 'travel time budget' allocated for travelling to work. Studies have shown that people are generally unwilling to commit more than 30 minutes to a frequently made travel trip [35,36]. A similar principle may be applied to daily physical activity. Individuals may have a 'physical activity time budget' not dissimilar to the current physical activity guidelines [37,38]. Thus, those new dog owners who perceive that taking their dog for a walk contributes to their daily exercise time may substitute dog walking in place of other types of physical activity. Nevertheless, the overall unadjusted effect of getting a dog on physical activity is positive and future research should explore the substitution effect, if any, of dog walking on other types of physical and sedentary activity.

While dog acquisition appears to positively influence the initiation of walking behavior, dog ownership may be more important for the maintenance of such behavior over time. For example, a study in the elderly found that after three years, dog walkers maintained their mobility advantage over owners who did not walk their dog and non-owners [11]. A dog may facilitate an increase in physical activity through its positive effect on owner's cognitive beliefs about walking and by providing motivation and social support for walking [13], however the long term nature of the dog-owner relationship suggests that dogs have a more significant role to play in terms of maintenance of physical activity. The characteristics of dog walking are similar to a number of factors shown to be associated with adherence to physical activity [39-42]. Walking the dog is relatively easy and of moderate intensity, it can be incorporated into daily life, and it is enjoyable since the dog is often considered a family member [43,44]. Moreover, it provides contact with nature [45], can facilitate sense of community and social capital [46-49] and over the lifespan of the dog (usually several years) provides owners with a daily extrinsic and intrinsic cue to be active.

In addition, dog ownership may help with the maintenance of physical activity behavior during periods of transition. For example, in this study, dog acquisition was associated with people moving from a single to couple relationship. Previous research has shown that changing from a single lifestyle to cohabitation is associated with changes in health-related behaviors such as decreased physical activity, poorer dietary habits and weight gain [50-52]. People who own a dog during important life transitions may be better equipped to maintain their walking behavior because the dog's enthusiastic wagging tail provides its owner with a positive cue to be active. Moreover, dog ownership may encourage new family members to take up physical activity through dog walking.

### Study limitations

This study's large sample size with sufficient power to examine the association between dog acquisition and increased recreational walking is a strength. However, generalizability of the findings may be compromised because RESIDE study participants are moving into new housing developments and may not be representative of all new dog owners and continuing non-owners. Moreover, this study relied on self-reported physical activity and it is possible that new dog owners may have over-estimated the minutes spent walking with their dog. Future studies would be strengthened by objectively measuring dog walking behavior. Finally, this study was not able to ascertain the causal pathway between dog acquisition and increase in intention to walk. Reasons for acquiring a dog, type of dog acquired and age of dog at follow-up are relevant factors to consider when investigating whether dog acquisition increases walking and should be considered in future prospective studies. Furthermore, it may be useful to examine the effect of long term intention (e.g. intention to walk in the next 6 months or 12 months) on the relationship between dog acquisition and walking.

## Conclusion

Our study provides longitudinal evidence to suggest that dog acquisition leads to an increase in walking. Dog acquisition increased recreational walking by 31 minutes/week and this relationship persisted after adjusting for baseline recreational walking and baseline factors associated with dog acquisition. Moreover, increased intention to walk mediated the relationship between dog acquisition and increased recreational walking. It is likely that the mechanisms through which dog acquisition facilitates increased physical activity is through behavioral intention via the dog's positive effect on owner's cognitive beliefs about walking and from motivation and social support for walking. Furthermore, while it appears dog owners may substitute dog walking for other types of physical activity; it is likely that the long-term commitment of dog ownership plays a significant role in assisting owners to maintain their walking behavior. Considering that 40% of households in the United States and Australia own a dog, examination of the effect of dog ownership on physical activity adoption and adherence warrants further investigation.

## Competing interests

The first author (Hayley Cutt) is supported by an Australian Research Council, Australian Postgraduate Award – Industry which has Petcare Information and Advisory Service as the Industry Partner. The Petcare Information and Advisory Service placed no restrictions on the design, analysis, interpretation or publication of study findings. All other authors declare that they have no competing interests.

## Authors' contributions

HC conceived and designed the study and, analyzed and interpreted all data. HC drafted the manuscript revising it critically at each stage. MK advised on the design and analysis of the study, the interpretation of results and provided input at each stage of the manuscript draft. BG-C advised on interpretation and implications of results also providing input at each stage of the manuscript draft. All authors read and approved the final manuscript.
